# Africa’s contribution to global sustainable and healthy diets: a scoping review

**DOI:** 10.3389/fnut.2025.1519248

**Published:** 2025-05-02

**Authors:** Ruth Oniang’o, Zadok Maingi, Silvester Jaika, Silvenus Konyole

**Affiliations:** ^1^Professor of Food Science and Nutrition, Executive Director-Rural Outreach Program and Editor-in-Chief, African Journal of Food, Agriculture, Nutrition and Development, Nairobi, Kenya; ^2^Department of Food Science, Nutrition, and Technology, School of Health Sciences, South Eastern Kenya University, Kitui, Kenya; ^3^Department of Health Sciences, Rift Valley Technical Training Institute, Eldoret, Kenya; ^4^Department of Nutritional Sciences, School of Public Health, Biomedical Sciences, and Technology, Masinde Muliro University of Science and Technology, Kakamega, Kenya

**Keywords:** African diet, Western diet, food security, nutrition transition, food systems, Africa

## Abstract

**Background:**

A healthy diet is essential for human wellbeing and environmental sustainability. Africa possesses diverse traditional food systems that are nutritionally rich and environmentally sustainable. However, modern dietary transitions and increasing reliance on imported and processed foods threaten the continent’s food sovereignty and public health. This review explores Africa’s contributions to healthy diets and sustainable food systems.

**Objective:**

To examine the role of Africa’s traditional diets in promoting global health, and to assess the impact of dietary transitions on nutrition and food security.

**Methods:**

A scoping review was conducted using PubMed, Scopus, Web of Science, Google Scholar and some information from FAO repositories. Studies published between 2015 and 2024 were included, with some earlier studies providing historical context. Thematic analysis was used to synthesize findings on African diets, dietary transitions, and global contributions.

**Findings:**

Traditional African diets are rich in whole grains, legumes, vegetables, and fermented foods, offering high nutritional value and health benefits. Dietary transitions toward Westernized diets have led to increased consumption of processed foods thus contributing to rising rates of obesity and non-communicable diseases. Africa’s indigenous foods, such as sorghum, millet, teff, amaranth, and baobab, are gaining global recognition for their health benefits. Sustainable food systems in Africa present solutions for addressing global food security challenges.

**Conclusion:**

Africa’s traditional food systems provide valuable insights into healthy and sustainable diets. Promoting indigenous African foods and preserving traditional dietary practices can enhance global food security and nutrition. Policies and investments should focus on revitalizing traditional African diets to address nutrition and food security challenges.

## Introduction

**Figure 1:**
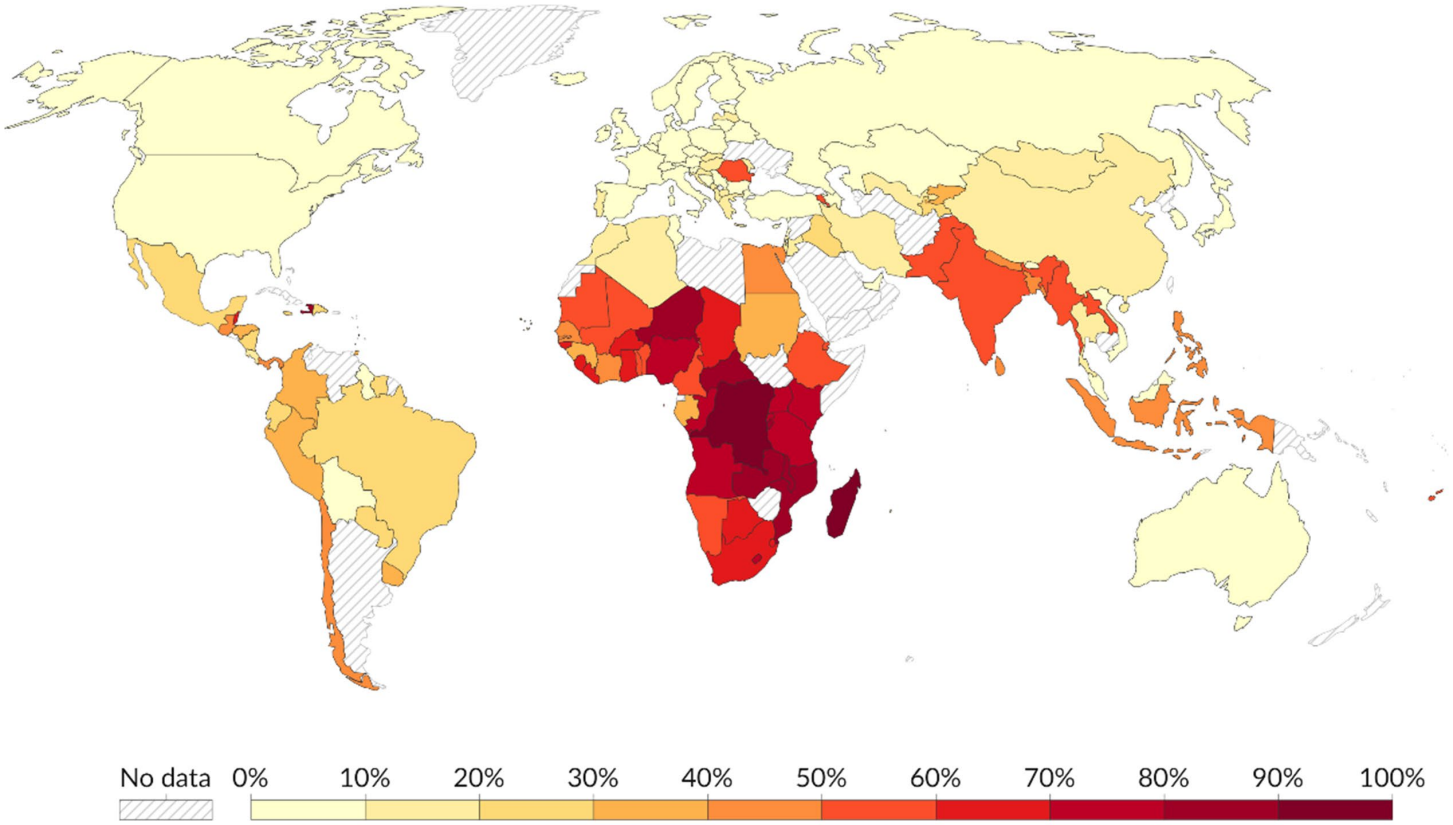
Global distribution of the population that cannot afford a healthy diet in 2022. Data source FAO and World Bank (106), licensed under CC BY 4.0, using data and methods from Herforth et al., (110), licensed under CC BY-NC-SA 3.0 IGO.

The World Health Organization (WHO) defines a healthy diet as one which provides all the essential nutrients required by the human body to support an individual’s physical and mental wellbeing (1). The description of a healthy diet is deduced from the Food and Agriculture Organization (FAO) 1996 World Food Summit in Rome food security definition which stated that food security exists when all people, at all times, have physical and economic access to sufficient, safe and nutritious food that meets their dietary needs and food preferences for an active and healthy life (2, 3). A healthy diet consists of a variety of foods that deliver the essential carbohydrates, proteins, fats, vitamins, minerals, antioxidants and fiber while minimizing the intake of harmful substances such as excess sugars and salt, saturated fats and highly processed foods (4). A healthy diet emphasizes the adequate consumption of whole grains, plant and animal- based proteins, healthy fats, and water (5). Globally, the World Economic Forum (WEF) estimated that 3 billion people cannot afford a healthy diet (6, 7). The FAO shows that nearly three-quarters of the African population cannot afford a healthy diet (Figure 1) and more than half cannot afford a nutrient adequate diet (8). The increasing food imports into Africa has worsened the situation and the resultant food importation bill is about $35 billion which is estimated to rise to $110 billion by 2025 (9). This weakens the African economies and lowers agricultural production.

The high food prices, low income levels and some consumer preferences have been reported as major barriers to affording healthy diets in some African countries (10, 11). In some of the African countries like Angola, the cost of a healthy diet is as high as USD 4.5, in Sudan USD 4.3 and for both Nigeria and Guinea it is USD 4.1 (7). Households which cannot afford the least-cost healthy diet in their countries are likely facing some degree of food and nutritional insecurity and thus face the risk of child and adult malnutrition (12). The demand for food in Africa is increasing with the increase in population and the intensifying climate change impacts and thus improving the agricultural infrastructure will be crucial to produce affordable foods to the population (13, 14). The State of Food Security and Nutrition in the World, 2024 emphasizes the more innovative investments in agrifood systems to ensure that households can access and afford a healthy diet (15). Therefore, the review aims to explore Africa’s contribution to global dietary health and environmental sustainability.

## Methodology

### Review approach and justification

This review article synthesizes existing literature on African traditional diets, comparisons with the Western diet, evolution of Africa’s food sources and dietary transitions and the implications of African diets on global health and sustainability. The review methodology employed a narrative and scoping review to ensure a systematic and comprehensive approach to articles selection and data synthesis. This approach allows for the synthesis of diverse literature sources such as empirical studies, policy documents and historical analyses thus providing a broad and structured understanding of the topic.

### Search strategy

A structured literature search was conducted from PubMed, Scopus, Web of Science and Google Scholar databases. A further search was conducted from the FAO repositories since they provide authoritative data on food composition, dietary transitions, food security and policy recommendations relevant to African traditional diets and their global implications. Foundational studies relevant to traditional African diets were also considered. The search was restricted to studies published between 2015 and 2024 to ensure the inclusion of recent empirical research. However, some papers published before 2015 that provided essential historical context relevant to understanding dietary transitions in Africa were included. The keywords and Boolean operators used in the search were: (“African traditional diets” OR “indigenous African foods”) AND (“nutrition” OR “health benefits” OR “dietary transitions”), (“African food systems” OR “African food sources”) AND (“historical trends” OR “food evolution”), (“Western diet” OR “modern diets” OR “processed foods”) AND (“health effects”), (“African diet” AND “Western diet”) AND (“nutritional comparison” OR “health impact”), (“African diet” OR “traditional African foods” OR “indigenous African nutrition”) AND (“Western diet” OR “modern dietary patterns”) AND (“nutrition transition” OR “health outcomes” OR “sustainability”).

### Article selection

**Figure 2:**
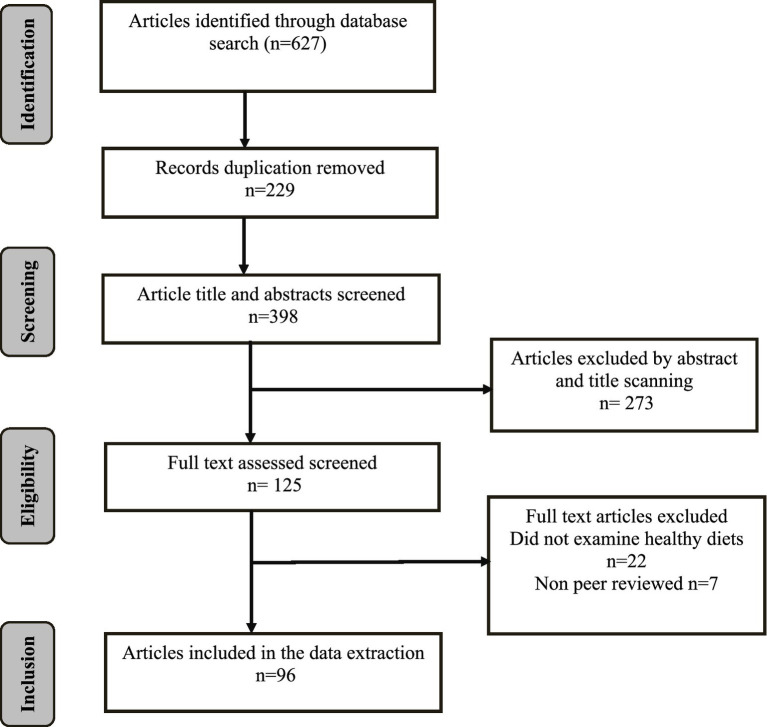
PRISMA flow diagram of article identification and selection.

Following recommended protocols for scoping reviews, at least two reviewers were involved in the abstract and full text screening of each article in order to minimize reporting bias (16). The database search resulted in 627 articles. After removing duplicates, 398 articles remained. The initial round of title and abstract screening yielded 125 eligible articles. A further round of full-text screening resulted in 96 original articles for inclusion in this review (Figure 2). There were no conflicts between independent reviewers regarding the eligibility of articles for inclusion.

### Inclusion and exclusion criteria

The selection of studies for this review was guided by defined inclusion and exclusion criteria designed by the authors to ensure relevance and quality.

*Inclusion criteria*: we considered peer-reviewed journal articles, government reports and policy documents published in English that specifically address African diets and nutrition. The review prioritized research articles that presented comparative analysis of African traditional diets with Western diets. Additionally, studies that provided empirical data on dietary transitions, nutrient composition and health impacts were also included.

*Exclusion criteria*: the review excluded articles that lacked primary data or were not systematic reviews of existing literature. Studies that focused exclusively on food processing technologies without addressing dietary impacts were also excluded.

### Data extraction and synthesis

The thematic data extraction and synthesis focused on the composition of the African traditional diet, historical dietary evolution, and shifts influenced by modernization and globalization. Comparisons between the African and Western diets were drawn to highlight their nutritional value, health impacts and environmental sustainability. The review also examined the challenges posed by modern dietary transitions. Additionally, it explored Africa’s contributions to global nutrition and sustainability through indigenous food systems and eco-friendly agricultural practices. Finally, the synthesis identified the solutions Africa offers to improve both local and global dietary health. This provides key insights through thematic categorization, comparative analysis and conceptual mapping to highlight knowledge gaps and research opportunities.

## Results and discussion

### Articles reviewed by thematic area

The review included a total of 96 articles, categorized into six thematic areas based on their focus. Thirty-one articles examined the evolution of Africa’s food sources, outlining the past dietary trends and contemporary shifts. Twenty-three articles explored the composition, nutritional value and cultural significance of a typical African diet. Twenty-two articles covered the comparisons between the African diet and the Western diet. These articles provided insights into key differences in food sources, processing methods and health implications. The Western diet was analyzed in seven articles that primarily focused on its characteristics and associated health risks while eight articles discussed the challenges of modern diets. Lastly, five articles examined the global contributions of the African diet to healthy diets and solutions Africa has for itself and for other continents.

### The typical African diet

The traditional African diet varies widely across the African regions due to food production, consumption and cultural patterns. However, the African diet is generally characterized by reliance on starchy foods such as maize, millet, sorghum, cassava, and yams complemented by leafy vegetables, legumes, nuts, seeds and fruits (17). The cereal based starchy foods made from maize, sorghum, millet and wheat are most consumed in the form of porridge, *ugali* (a stiff porridge made by mixing cornmeal with boiling water) and bread while the tubers are boiled, roasted or fried. The most widely consumed African indigenous green leafy vegetables include: amaranth (*Amaranthus spp*), spider plant (*Cleome gynandra*), jute mallow plant (*Corchorus olitorius*), pumpkin leaves (*Cucurbita spp*), African nightshade (*Solanum spp*), nettles (*Urtica massaica*) and cowpea (*Vigna unguiculata*) (18, 19, 101). The ALVs though underutilized have great nutritional and medicinal value to humans, for example jute mellow provides antioxidants to the body while other vegetables are rich in B1, B2, C, and carotenoids, and minerals (20, 21). Amaranthus is rich in carotenoids (9.0 ± 0.2 mg), vitamin C (43.0 ± 1.6 mg) and lutein (14.7 ± 0.8 mg) and Retinol Activity Equivalent (RAE −0.8 ± 0.02) when raw; these nutrients have anti-inflammatory role in the human body (22, 23). Some animal products such as fish, meat, fermented milk, poultry, beef and mutton and to a small extent game meat also dominate the African diet (24). However, these animal proteins are consumed less frequently in some areas due to economic or cultural reasons, and this makes the African diet naturally lower in fats (25, 99). There are communities which, by culture, take considerably more meat and milk, such as the Masaai in Kenya and Tanzania (104, 105).

West African cuisine is majorly composed of rice, millet and sorghum as main staple foods which are served together with plenty of vegetables along with a variety of spices and seasonings for flavor (26). However, the increasing intake of dietary energy, fat, sugars and protein and low consumption of fruit and vegetables in West Africa represents a critical nutrition transition (27). The diet also consists of cassava and yams as the main staple tubers (28). Beans (*Phaseolus vulgaris*), black-eyed peas (*Vigna unguiculata*) and peanuts (*Arachis hypogaea*) also feature prominently as the main legumes in the West African diet. The significant sources of proteins in the West African diet includes dried or smoked, poultry, goat or beef (29). The diet depends on access to diverse food sources and is rich in complex carbohydrates, fiber and vitamins but it can sometimes be low in protein and essential micronutrients (30). In other words, legumes for many communities have been the main providers of proteins, and therefore mostly plant proteins.

The diet in Central Africa comprises diverse plant and animal products that reflect the region’s agricultural practices, geographical diversity and cultural traditions. These include cassava and other tubers such as yams and sweet potatoes, while the grains include millet, maize and sorghum. People also eat plantains as the main carbohydrate source (31). Some traditional staple vegetables include the dark green leafy vegetables such as amaranth and cassava leaves. The preparation of protein- rich legumes offers Central African populations an important protein source. Animal proteins are from fish, poultry, goat, beef and among others, and are less commonly consumed in low and middle income African countries due to cost or availability (32, 100).

The East African diet is normally composed of high intakes of minimally processed foods and most of the foods are cooked through boiling, steaming and fermentation. The East African diet is dominated by cereals like maize, sorghum and millet, tubers and legumes-based food products (33). In addition, beans, peas and lentils are important sources of protein while vegetables such as kale, spinach and other indigenous green leafy vegetables such as amaranth, jute mallow, cowpea and pumpkin leaves are rich sources of vitamins and minerals in the diet. Uganda has a unique cuisine of bananas and plantains which are consumed as either boiled, roasted or fried and *ebinyebwa* (Ugandan groundnut stew) (34). Most protein in the East African region comes from fish, poultry, beef or goat but it is sparingly consumed in some areas because of the high cost of purchase (35). Fruits include mangoes, oranges, pineapples and papayas, and dairy products are usually obtained from the pastoral communities, particularly in Kenya and Tanzania. The East Africans’ regard of processed foods is traditionally generally low but is gradually changing due to factors such as urbanization and the adoption of western diets (36).

Traditional and novel food interweave the typical Southern Africa diet citing the region’s culture (37). The staples in the Southern Africa region are based on maize and sorghum. Meat barbecues originating from beef, lamb, chicken and pork among others are commonly consumed in this region (38). They also take different vegetables such as pumpkins, potatoes, spinach and cabbages. The traditional diets include legumes and indigenous leafy greens such as amaranth leaves, spider plant, cowpea leaves, and African nightshade. The increased trend towards the Western way of living has resulted in a change in diet in the Southern Africa Region due to the influence of urbanization and economic development (39). However, the food systems transformation pathways in the Southern Africa region encourage consumers to take healthier traditional meals in spite of such changes (40).

The North African diet is characterized by a rich blend of flavors, ingredients and culinary traditions. Some products which can be considered staples include: couscous, semolina and bread from wheat or barley (41). Lentils (*Lens culinaris*) and chickpeas (*Cicer arietinum* L.) are the most widely consumed legumes which are accompanied by tomatoes, eggplants, peppers and others with animal protein. Most of the commonly consumed meats are lamb, chicken and beef while seafood is usually found in the coastal regions. Majority of the foods identified for preparation are cooked with olive oil and spices including cumin, coriander and saffron among others. Dairy products are also important food components. Some of the fruits include date fruits, figs and citrus which are usually eaten as snacks or dessert. Some traditional fermented foods as *injera* from Ethiopia [made from teff (*Eragrostis tef*), a tiny, gluten-free grain native to the Horn of Africa], contain natural sources of probiotically active substances that influence the state of gut microbiota (42–44). Some of the ingredients for the North African cuisine are produced locally and, therefore, are affected by seasonal changes.

### Evolution of Africa’s food sources: past trends and contemporary shifts

Historically, during the pre-colonial period, African food systems were highly localized, built around native crops cultivation, foraging for wild plants, hunting and pastoralism (45). Communities relied on traditional methods of farming, where both crop cultivation and animal husbandry were integral parts of their food systems (109). Smallholder farmers grew resilient crops like millet, sorghum, cassava, yams, and green leafy vegetables, while raising livestock such as cattle, goats, sheep, and poultry (46). Foods and medicines were also sourced from the wild and included honey, fruits, birds and game meat. These practices ensured that Africa fed herself with a balanced diet of nutrient-rich plant and animal foods. Food production in Africa remained at subsistence level and the farming system was based on shifting cultivation and bush fallow farming. Under these practices, soil fertility was maintained by opening fresh cultivation ground thereby allowing the most recently cultivated land to rejuvenate (18). Farmers applied organic manure once in a while and chemical fertilizers were not even known. Likewise, animal production was by pastoralism where herders migrated from one area to another in search of pasture land usually after every rain season (107). The combination of crops and livestock allowed healthy diets and diversified nutrition. The crops provided carbohydrates, vitamins and fiber, while animals offered essential proteins, fats, and micronutrients which are key for optimal nutrition status. The indigenous leafy vegetables also served as medicine.

It is estimated that Africa is comprised of about 30,000 species of edible plants which, out of these, about 7,000 are traditionally consumed (47). This large wealth of genes in agriculture and food production is a clear representation of the great ecological base of the continent and the possibility to achieve food security in the region (48). Africa’s rich bio-diversity demonstrates the ability to support a variety of food production systems with proper utilization and management of the indigenous plant and animal genetic resources. African countries have the resource endowment needed towards attaining food security and sustainable agriculture (109). With the evolution of society and agriculture, numerous foods that once shaped diets and cultural identity across Africa have been gradually displaced (49). Many of these crops are now considered neglected and underutilized species, having fallen out of mainstream agricultural and dietary practices despite their historical significance and nutritional benefits. Currently, 60% of African food is based on wheat, maize and rice (47). The change could be linked to the Green Revolution of the 1950s and 1960s that focused on monocrops like maize, wheat and rice, grown on a mass scale. Monocrops did not translate to success of food systems in Africa but undermined small scale farmers that ensured sufficiency of traditional foods, making food productions unsustainable (50). The much acclaimed Green Revolution made sense at the time as it was feared masses would starve.

African food self-sufficiency has changed significantly over the last five decades (47). Today, Africa finds itself increasingly dependent on imported foods, a shift driven by global economic forces, changing diets, and urbanization (46, 102). While agriculture remains a dominant part of the economy, the continent’s focus has further shifted from producing food primarily for local consumption to exporting crops like tea, coffee, cocoa, and flowers, to generate much needed foreign exchange. This emphasis on cash crops, grown mainly for international markets, has weakened Africa’s ability to feed her own population. Staple foods that were once widely produced locally in Africa such as millet and sorghum are now often imported (51). A recent report by the United States Department of Agriculture (USDA) foreign agricultural service showed that South Africa Alone was estimated to import about 40,000 metric tons of sorghum to meet the local demand (52). Maize that was introduced and adopted in Africa from Mexico in the early 20th Century is also currently being imported since most of the maize production in Africa is done under rain-fed conditions (53), and cannot meet increasing consumer demand. Africa imports 28% of its required maize grain from countries outside the continent and according to Famine Early Warning Systems Network (FEWS NET) in the 2023/2024 year alone, maize imports in Africa exceeded 2 million metric tons (54). Imported rice, wheat, processed foods, and frozen meats have become common across Africa especially in urban areas with the growing populations. The shift has impacted negatively on the access of traditional, health foods in many African countries and increasing the reliance on calorie dense and over processed food posing a risk for lifestyle diseases (WHO, 2023).

Africa spends US$78 billion annually on food imports, with some countries like Zimbabwe, Guinea, and Sudan exceeding 100% of their foreign currency earnings on these imports (55). According to the African Development Bank, the continent’s food and agriculture market, valued at US$280 billion in 2023, could rise to US$1 trillion by 2030 with strategic investment (56). The trend in the level of food imports and exports is one of the areas that underlines a marked change in African food systems (57, 58). Sustainable organic farming that was the mainstay of the traditional agricultural system is progressively being substituted by monoculture and commercial farming. Such contemporary systems are inclined toward the production of export crops which deprive people their sovereignty right over the kind of foods they consume. As a result, it is concerning that African countries have become reliant on imports for a sizable percentage of the food they eat today with much of it consisting of ultra-processed foods that do not provide the same nutritional benefits as was once the case (103). This change of diet not only reduced dietary diversity but also has led to an epidemic of non-communicable diseases, as processed, calorie-dense foods have replaced whole nutrient-dense foods, eradicating the nutritional bulwark of Africa.

Compounding this problem is the forces of climate change which have destabilized food production across the continent. Various changes in environmental conditions such as long dry seasons, irregular rainfall and high temperatures have had negative impacts on agricultural production and rearing of livestock, respectively (59). An analysis of countries in sub-Saharan Africa show that, an increase in the temperature by one degree lowers the value of agricultural production. Households that engage in diverse farming activities are better equipped to handle high temperatures. This adaptability reduces the negative impacts of climate change and helps these households build resilience, especially in areas that rely on rain-fed agriculture (60, 98). These climate changes have led to low productivity and food insecurity. Farmers have lost earnings because they cannot adapt to change and many have had to ditch crops which were clearly suited to their local climate conditions (61). The level of imported foods has risen as a result of the low yields, thus degrading food sovereignty in Africa.

Huge changes are being observed through the process of urbanization in Africa through a shift in diets towards processed and convenience foods (62). One of the impressions that cities give is that when people leave rural places to acquire residence in the urban centers, they leave behind customized production practices and knowledge of how to feed the world. In the urban areas, fast foods, refined grains, and sugary beverages dominate the market resulting in high consumption of processed foods. This not only changes the trend of meal taking, but also continues pulling away Africa from its farming base by discouraging indigenous food practices (63). In Burkina Faso, where participants from both urban and rural areas were compared, the researchers found that the urban group consumed more animal protein and simple sugars. However, the group of rural and semi-urbanized people consumed significantly more fiber (64). This is in conformity with the general changes in dietary habits of urban people from traditional diets to processed foods.

There is still an issue for the agricultural systems to meet the standards of modern technologies and most African countries still use labor-intensive farming techniques. Mechanization that is a potential to augment productivity remains wanting in most parts of the continent owing to costs and infrastructure (49). Agricultural practices rooted in hoe and plough mean that farmers’ technologies are incapable of meeting food demands that come with a more urbanized population. The late industrialization of mechanization reduces agricultural productivity and increases Africa’s food systems’ susceptibility to climate change and population trends (65).

Another area of controversy is genetic engineering of foods. Where some believe that adopting genetically modified (GM) crops is the solution to the food security challenges facing Africa due to the increased crop yields, tolerance to pests and droughts many remain skeptics (66). Misconceptions relating to GM foods remain prevalent, coupled with apprehension that the genetically modified crops may have deleterious effects on health, environment or that this makes farmers heavily dependent on multinational companies for seeds and other production necessities (67). Most African nations have approached GMOs with apprehension, fearing the dangers they present to agriculture and the natural foods world. But some have considered it timely, economical and immune to climate spikes and have been making incomes out of it. This means that if the fears are brought to task, GMO could be the food solution for the next generation (113).

Cultural factors also play a significant role in shaping Africa’s food systems, and there is a need to understand cultural dimensions in the realignment of food systems in Africa. In many communities, the local population has increasingly shifted its palate towards consuming foods that are marked as “imported” or “western” pending their social-economic status (63). This shift in culture has seen the dumping of traditional food patterns which were so relevant to the diets of Africa (50). Locally grown crops, vegetables and naturally raised animals are now increasingly losing out to easily available processed foods, milled grains and commercially produced meats. As these traditional foods are replaced with western foods in our society and people’s diets, so is a wealth of relevant information on how to preserve, process, prepare, and consume food in a way that is healthy and sustainable (68).

There is renewed effort to promote traditional food systems in Africa due to the difficult factors mentioned above. Currently, there is a trend to support and produce near-shore crops, use of organic products and appropriate agricultural soil for local production than the foreign products (112). Such a strategy is being promoted by advocates of food policies that cover issues ailing small- holder farmers and policies that promote consumption of traditional crops which are healthy and sustainable to the environment than the processed foods (64). African governments should also be able to develop national bio-economy policies to help appreciate natural resources for human and animal health and for the conservation of mother nature.

### The Western diet

The Western diet is characterized by a high intake of animal proteins, refined sugars and saturated fat (69). It also consists of natural and artificial food additives due to the presence of ultra-processed food and high amounts of refined salt. Fiber intake in the Western diet is often inadequate due to a lack of whole grains and legumes (70). The emphasis on convenience over quality in many Western countries has led to a disconnection from the source of food, making it difficult for people to trace the origins of what they are consuming. Excessive consumption of red meat, dairy products and sugary beverages has contributed to a wide range of health issues including obesity, heart disease, diabetes and other chronic conditions (71, 72). The high consumption of refined sugars and salt has led to public health concerns over hypertension and metabolic disorders (73). Western diet is said to lead to dysbiosis, with a decreased richness and diversity of total bacteria with a reduction in numbers of beneficial microbiome and an increase in the harmful ones in comparison with a plant-based diet (74). The detrimental effects of the Western diet on gut microbiota may also be driven by food additives inducing dysbiosis and consequently adverse intestinal mucosal effects and inflammation (108).

### Comparisons between the African diet and the Western diet

The African diet significantly differs from the Western diet in several aspects such as the nutritional composition, the sources, consumption trends, processing methods, among others. The information in Table 1 shows how the two diets compare.

**Table 1:** Major comparisons between the African diet and the Western diet.

Aspect	African diet	Western diet
Nutritional composition	- High in plant-based foods such as whole grains (millet, sorghum, maize), legumes (beans, lentils), vegetables, fruits, and tubers (cassava, yams) (81)	- High in processed foods, refined sugars, and unhealthy fats (saturated and trans fats) (69).
	- The indigenous African diet is characterized by vegetables, wild fruits, lean meats, legumes, and staple starches with high fibre and phytochemical profiles (82)	- Low in fiber, fruits, vegetables, and whole grains with fiber marketed as a single product (69)
Food sources	- Food production is usually from localized, small-scale farming indigenous crops with high diversity across regions and food availability is affected by seasonal variabilities (50, 83)	- Large-scale industrial agriculture producing monoculture crops (corn, wheat, soy) (84)
	- Traditional diets are sourced from natural and less industrialized environments although urbanization is increasing processed food consumption (85)	- High reliance on processed, pre-packaged and convenience foods (74)
Cultural context	- Deeply embedded in cultural traditions and rituals and food is often shared communally with an emphasis on family and social connections (86)	- Less emphasis on communal eating. Food choice is driven by convenience with a fast-paced lifestyle influencing food choices (87)
	- Traditional cooking methods include boiling, fermentation and drying which preserve nutrients and enhance their bioavailability (24)	- Most commonly used cooking methods include frying, grilling and baking which may destroy proteins and vitamins at high temperatures (88)
Dietary trends and transition	- Rapid nutrition transition in urban areas with increased consumption of Westernized diets and processed foods (89) - Urbanization is driving a transition toward Western diets with increased consumption of fast food, sugary beverages and processed products. - There is a growing interest in revitalizing traditional African foods, particularly indigenous grains like millet, sorghum, and teff, to improve food security and nutrition (91, 92)	- Already fully industrialized, with minor shifts toward healthier and plant-based diets in response to public health campaigns and consumer demand (90)
		- Emerging health-conscious movements promoting plant-based, organic, and whole-food diets. - Growing popularity of vegetarian, vegan and flexitarian diets (93, 94)
Health concerns	- Historically associated with lower rates of chronic diseases in rural areas where traditional diets are predominant. However, starchy diets commonly consumed in Sub-Saharan Africa often lack various micronutrients, including iron, zinc, calcium, folate, iodine, vitamin A, and vitamin B12. This poses the risk of triple burden of malnutrition with undernutrition and micronutrient deficiencies being prevalent in food-insecure areas while obesity and non-communicable diseases rates rising in urban areas (30, 95)	- The diet is usually calorie-dense, nutrient-poor, leading to increased risks of chronic and non-communicable diseases such as obesity, heart disease, hypertension and diabetes due to excessive consumption of ultra-processed foods, high sugar intake, unhealthy fats and sodium. This has raised concerns over high mortality rates originating from these diseases (96, 97)

Source: researchers review of literature.

### The challenges of modern diets

One of the biggest challenges of the present day diet in different countries is an insufficient consumption of fiber (62). The traditional African diets included vegetables, legumes and whole grains most of which provide dietary fiber that is important for good digestion (111). Fiber has confirmed obligations when it comes to bowel movement, avoiding constipation, and promoting gut health. Such fiber-rich foods have for a long time been linked with decreased chances of developing several chronic disorders such as heart disease, obesity, as well as type 2 diabetes and colon cancer.

However, the changes in food habits from whole grains and high fiber products to refined carbohydrates, processed foods and soft /sugar sweetened beverages have essentially removed this component, making fiber intake way below today’s standard. Current foods include over processed foods like white bread and pastries that in the process of refining are deprived of fiber (69). Further, it noted that the consumption of foods such as cakes and soft drinks is now frequent and yet fiberless. This change in diet has some serious ramification for health as pointed out by Akinola et al. (50). The effect of low fiber diet is gradually emerging, which poses numerous health risks. A lack of fiber can also cause minute injuries or inflammation to the intestines, which result in constipation and other gastrointestinal illnesses. Fiber helps to fill the colon and, therefore, assists in its functioning; in its absence, people develop conditions as diverticulitis, hemorrhoids, among others. Also, foods rich in fiber contribute greater satiety or will power, and help to ease the weight problem. Diets devoid of fiber may lead to over-consumption of food and lead to weight gain, unlike when fiber is consumed.

Excessive sugar and refined salt are pervasive in today’s diets. These additives are found in processed snacks, cereals, and drinks, contributing to high blood pressure, diabetes, and cardiovascular diseases (64), all conditions that are alien to Africans. Furthermore, the over-processing of food destroys its nutritional value. Many of us now consume food that is far removed from its natural state (18). Packaged and processed items are loaded with preservatives and additives, making it hard to even recognize what we are eating. The world is at a critical juncture, and Africa has a great deal to offer. There is need for a roadmap to reversing the damage caused by over-processing and unhealthy dietary habits by reverting to the traditional African food systems rich in minimally processed or unprocessed plant-based foods (75, 76).

### Global contributions of the African diet to healthy diets

Nutrient-dense foods such as cassava, yam and sweet potatoes in the African diet contribute to the vital calories. Sorghum and millet, containing fiber, B vitamins, iron, and zinc, are global contributions for better grain substitutes. A grain called Teff, now referred to as a “superfood” from Ethiopia and packed with protein, calcium, and iron, joins the world diet to meet the gluten intolerance demand. Beans and pulses such as cowpeas, pigeon peas, and bambara groundnuts are vital plant sources of protein for Africa and provide valuable fiber and lower cholesterol levels. They are a source of protein, healthy fats, and antioxidants and fit well with global trends for healthier snacking and the inclusion of pulses in diets.

Vegetables like amaranth, moringa, and pumpkin leaves contain significant amounts of vitamins A and C, calcium, and antioxidants (19, 77, 78). African foods such as okro, which is very common in the West African region, are rich in fiber and hence ensure better digestion. They reinforce the Western Africa dietares’ recommendations by the global nutritional policy to eat more vegetables. New-age grains and millet from the African continent are becoming known for their health-enhancing qualities. They are richer in dietary fiber, have lower glycemic values, are normally low in gluten, and are more nutritious than processed grains. Maize, which is prevalent in Africa, also avails important nutrients and fiber to the world’s food basket when consumed in its whole grain form to enhance wholesome eating.

Bread from teff known as Injera, fermented maize known as “Kenkey,” and sour milk are examples of fermented probiotic foods well known in sub-Saharan Africa. These traditional foods match up with the current global desire for fermented foods for the wellbeing of the gut biome, thus promoting society’s value for antibiotic-free digestive health in natural unadulterated cultured foods. Foods such as red palm oil and baobab oil found in Africa are good sources of fats that are healthy and contain antioxidants, which when taken in moderation, are good for the cardiovascular system. Shea butter is well known in the Central and West African Countries such as the Central African Republic, Cameroon, Nigeria, Benin, Burkina Faso, Mali, Ghana, and Guinea, and its stock is becoming valuable as it is used for cooking as well as in therapeutic services (79, 80). Collectively, these oils align with current global tendencies and accredit healthier and naturally occurring sources of fat nutrients. Innovative exotic fruits that are specific to Africa, such as baobab, marula and tamarind, contain vitamins, especially vitamin C and various antioxidants, hence qualifying for the growing global market for superfoods and healthy snacks. Staple items like plantain and bananas provide potassium and other nutrients to supplement other food items; their consumption greatly contributes to the nutritional requirement worldwide.

### Solutions Africa has for itself and for other continents

Africa must revert to diets that prioritize natural, whole foods, and high fiber intake while minimizing sugars, refined salts, and over-processed foods. The lessons of Africa’s traditional dietary wisdom could be one of the most powerful offerings to the global health movement, as we strive for healthier, more sustainable food systems. Current African diets are aligned to new global dietary patterns that include reduced consumption of meats and increased intake of dietary plant-based products like grains, pulses, and vegetables. This is not only good for the individual’s health but also for the environment. There is an appeal to people’s altruism that if they change their diet, they will not only become healthy themselves but also help save the planet as plant-based diets are considered to have a smaller negative impact on the environment.

These foods can be processed by steaming, grilling, or boiling; this drastically reduces the fat content while retaining nutrients in food. Essentials of Ghanaian foods such as jollof rice, *waakye* (Ghanaian dish made of cooked rice and beans), and kuku *paka* (made from grilled chicken, coconut milk, cream, and curry spices) incorporate natural foods and spices, yielding a nutritive value to the preparation and thus making a contribution to the knowledge of healthy preparation methods across the globe. Food systems across Africa are diverse and play a critical role in the preservation of global biological and ecological diversity. Baobab (*Adansonia digitata*), moringa (*M. oleifera*), and local leafy vegetables use minimal amounts of water and fertilizers since they adapt easily to adverse conditions and are native crops. It also underpins climate-smart and sustainable consumption of diets that are safe for human health and also friendly to the earth.

## Conclusion

In conclusion, the affordability of healthy diets for human consumption continues to be a great challenge in Africa due to the costs associated with it. Most of the traditional African foods that were both healthy and climate resilient have been neglected over time. There is a witnessed dietary shift to the energy dense convenient foods from the western diet due to cultural changes and increased urbanization. The advent of genetically modified foods continues to threaten the African traditional foods and the possibility of reversal trajectory remains unknown. The possibility of reverting to the traditional African foods remains a big debate with no solution in sight. However, we remain hopeful for more resources to go towards more research and sharing of knowledge.

The study findings highlight the need for policies that promote the preservation and integration of traditional African diets into national and global food systems. Governments should invest in research, education and public awareness campaigns to encourage the consumption of indigenous, nutrient-rich foods while reducing reliance on ultra-processed foods. Agricultural policies should support smallholder farmers to produce healthy traditional foods by improving access to resources, infrastructure and markets. Additionally, food security policies should prioritize sustainable agricultural practices that enhance resilience to climate change. At the global level, Africa’s contributions to healthy and sustainable diets should be recognized in international food policies, trade agreements and nutrition guidelines to promote food sovereignty and reduce the double burden of malnutrition across the African continent.
